# Withaferin A protects against hyperuricemia induced kidney injury and its possible mechanisms

**DOI:** 10.1080/21655979.2021.1882761

**Published:** 2021-02-10

**Authors:** Xia Zhao, Jing Wang, Liying Tang, Pei Li, Jing Ru, Yuzhi Bai

**Affiliations:** Department of Geriatrics, Beijing Chaoyang Hospital Affiliated to Capital Medical University, Beijing, China

**Keywords:** Withaferin A, potassium oxonate, hyperuricemia, xanthine oxidase, urate transporter

## Abstract

The study was designed to explore the effects of Withaferin A (WFA) on hyperuricemia-induced kidney injury and its action mechanism. Potassium oxonate (PO) was employed to establish the hyperuricemic mouse model. The pathological changes of renal tissue were evaluated by hematoxylin-eosin and masson trichrome staining. The levels of creatinine, blood urea nitrogen (BUN), uric acid (UA) and xanthine oxidase (XOD) were detected using corresponding commercial kits. Expressions of collagen-related and apoptosis-associated proteins in renal tissues were, respectively, evaluated by immunofluorescence and western blotting. Cell apoptosis was detected by TUNEL assay, and transporter expressions using western blotting. Followed by WFA, NRK-52E cells were treated with UA before evaluation of apoptosis and fibrosis. Results indicated that WFA ameliorated renal damage, improved kidney function, and decreased levels of creatinine, BUN, UA, and XOD in PO-induced hyperuricemic mice. Furthermore, WFA significantly prevented renal fibrosis and increased the expression of collagen-related proteins. Similarly, WFA markedly inhibited renal apoptosis, accompanied by changes of apoptosis-related proteins. Importantly, expression of transporters responsible for the secretion of organic anion transporter 1 (OAT1), OAT3, ATP-binding cassette subfamily G member 2 (ABCG2) was remarkably enhanced whereas that of urate transporter 1 (URAT1) and glucose transporter 9 (GLUT9) was reduced in renal tissues of mice with hyperuricemia. *In vitro* study revealed that WFA notably ameliorated UA-induced cell fibrosis and apoptosis. Taken together, WFA improves kidney function by decreasing UA via regulation of XOD and transporter genes in renal tubular cells.

## Graphical abstract

**Figure:**
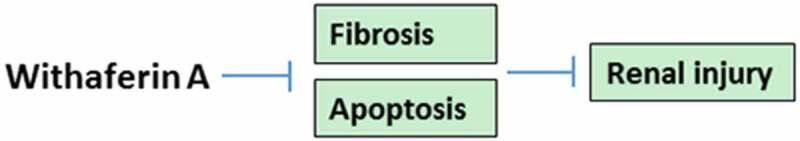

## Introduction

Hyperuricemia is featured by increased serum uric acid (UA) level [1], which leads to accumulation of urate crystals in the joints and further increases the risk of gouty arthritis, UA nephrolithiasis and other kidney diseases [2]. Elevated UA level is also reported to have prognostic significance in chronic kidney disease (CKD) and end-stage kidney disease (ESKD) [3,4]. Additionally, it has been generally acknowledged that urate nephropathy induced by hyperuricemia and hyperuricosuria can often contribute to acute kidney injury (AKI).

The occurrence of hyperuricemia is a result of imbalance between production and UA metabolism in renal tubules and intestines [5]. UA is the final product of purine nucleotide catabolism formed by xanthine oxidase (XOD), and thus the level of XOD takes part in the regulation of UA. In addition, UA is also regulated by various UA transporters. For example, urate transporter 1 (URAT1) and glucose transporter 9 (GLUT9) are responsible for renal reabsorption, while ATP-binding cassette subfamily G member 2 (ABCG2), organic anion transporter 1 (OAT1) and organic anion transporter 3 (OAT3) facilitate renal secretion [5,6]. GLUT9 and ABCG2 are expressed in intestines, and both of them participate in the homeostasis of UA levels, as evidenced by a recent study [7]. URAT1 mediates the reabsorption of 90% of the filtered urate, which functions as the dominant mechanism for modulating blood urate levels in the body. Existing study has shown that human URAT1 (hURAT1) defect is closely connected to the pathogenesis of hyperuricemia and gout [8,9]. Therefore, XOD and URAT1 are considered as important targets in the regulation of hyperuricemia and gout.

Allopurinol (ALP) is an anti-hyperuricemic agent that works by weakening the activity of XOD and suppressing UA concentrations in serum. It can also produce serious side effects, including skin rashes and allergic reactions [10] and gastrointestinal toxicity [11]. Therefore, it is of urgent need to identify a promising agent that effectively regulates UA levels and prevents hyperuricemia with minimum adverse effect. Withaferin A (WFA), isolated from Withania somnifera, has been shown to exert an anti-inflammatory effect via suppressing nuclear factor κ-B. For example, a study demonstrated that WFA attenuates palmitic acid-induced insulin resistance and dysfunction in endothelial cells by inhibiting inflammatory responses [12]. Other studies have shown that WFA can inhibit fat formation of 3T3-F442A cells and improve insulin sensitivity (resistance to diabetes), assisting in weight loss of obese mice [13,14]. Moreover, a previous study has proven that WFA reduces the inflammatory response of gout-induced arthritis mice [15]. Nevertheless, it remains unclear whether WFA exerts protective effects on hyperuricemia-induced kidney injury.

Potassium oxonate (PO) is often employed as the inducement for hyperuricemic mouse model, and it is also used in our experiments to explore the mechanism underlying hyperuricemia. In short, the objective of the present study was to investigate whether WFA can control serum UA level and thereby protect PO-induced hyperuricemic mice against renal function damage.

## Materials and methods

### Animals

Male C57/B6 specific-pathogen-free (SPF) mice (five-week-old) weighing 25–30 g were obtained from SLACS (Shanghai, China). Animals were raised in a controlled environment with temperature of 22 ± 2°C and humidity of 50 ± 5% under a fixed 12-h light-dark cycle. All mice were given free access to water and food. The animal experiment in this study was approved by Animal Care and Use Committee of Beijing Chaoyang Hospital Affiliated to Capital Medical University.

### Model establishment and drug treatment

The hyperuricemic mouse model was constructed via injection with PO, an urate oxidase inhibitor as described previously [16]. Animals were assigned into seven groups with 10 mice in each group: Control, High (10 mg/kg WFA), PO (300 mg/kg PO), PO+ALP (300 mg/kg PO+65 mg/kg ALP), PO+Low (300 mg/kg PO+3 mg/kg WFA), PO+Middle (300 mg/kg PO+5 mg/kg WFA) and PO+High (300 mg/kg PO+10 mg/kg WFA). PO was dissolved in 0.9% saline before being intraperitoneally injected into the mice once daily for 7 days. WFA (Sigma-Aldrich; Merck KGaA) treatment (prepared in 1% DMSO+99% PBS vehicle; injected intraperitoneally) was given every day to the mice of concerned groups. ALP (65 mg/kg) was orally administrated for 7 consecutive days starting from the day when PO was given. 2 h after last administration, mice were anesthetized with pentobarbital sodium by intraperitoneal injection. Blood samples were collected and then centrifuged. Serum and urine from each mouse were obtained and stored at −20°C. Kidneys were collected and stored at −80°C.

### Hematoxylin & Eosin (H&E) staining

After fixation in 4% paraformaldehyde overnight at 4°C, the kidney tissues were conventionally dehydrated, cleared, and waxed. Paraffin-embedded posterior segments were then cut into 5-μm-thick. The tissue sections were deparaffinized in xylene and rehydrated in a descending ethanol series. After H&E staining, the sections were dehydrated with graded ethanol and xylene. Observation of the slides was done with a light microscopy (Olympus Corporation) for histological examination.

### Masson trichrome staining

The deposition of collagen in the renal interstitium was determined by Masson trichrome staining. The 5-µm-thick sections were deparaffinized with graded ethanol together with xylene, followed by stained with Masson’s trichrome. Pathological changes in the kidney tissues and collagen fibers were observed under a light microscopy (Olympus Corporation).

### Evaluation of renal function

The levels of renal functional parameters including serum creatinine and blood urea nitrogen (BUN) were detected with kits obtained from Nanjing Jiancheng Bioengineering Institute (Nanjing, China) following the manufacturer’s recommendations. Additionally, fluorometric assay kit (Beyotime, Shanghai, China) was employed to detect the levels of UA and XOD activity in serum.

### Cell culture

Normal rat kidney epithelial cells (NRK-52E) were the product of China Center for Type Culture Collection (CCTC, Shanghai, China). Cells were maintained in DMEM (Gibco, Waltham, MA, USA) containing 10% FBS (Gibco, Waltham, MA, USA) in the incubator with 5% CO_2_ at 37°C. UA (Aladdin, Shanghai, China) was dissolved in PBS (Termo Fisher Scientific, Waltham, MA, USA) as a stock solution of 5 mg/ml and stored at 4°C in the dark until use. Different concentrations of WFA (0.1, 0.5, and 1 μM) were used to treat NRK-52E cells in the presence or absence of UA (50 μg/ml) for 24 h. Untreated cells were used as control.

### Cell viability assay

Cell Count Kit-8 assay (Dojindo Laboratories, Kumamoto, Japan) was employed to evaluate the viability of NRK-52E cells which were plated in 96-well plates with the density of 1 × 10^4^ cells/per well. After treatment with UA or WFA for 24 h, 10 μl of CCK-8 solution was added to each well. The optical density at a wavelength of 450 nm after 4-h incubation was determined in a microplate reader (Bio-Rad, Hercules, CA, USA).

### Terminal-deoxynucleoitidyl transferase-mediated nick end labeling (TUNEL) analysis

Cell sections and paraffin-embedded renal sections were stained with a TUNEL kit (Roche, Basel, Switzerland), and counterstained with DAPI for the nuclei. Cells were fixed in 4% paraformaldehyde and 0.1% Triton X-100 before staining. Integrated optical density (IOD) analysis was employed to indirectly reflecting the apoptosis.

### Immunofluorescence assay

The 5-µm-thick sections were baked at 60°C for 2 h, followed by deparaffinization in xylene and rehydration in graded ethanol. Subsequently, the sections were washed with water and blocked with BSA (Sigma-Aldrich; Merck KGaA). NRK-52E cells were grown on sterile glass slides for 24 h to reach 80% confluency and were then serum starved for 12 h. After treatment with UA and WFA, cells were immobilized with 4% paraformaldehyde, permeabilized with 0.1% Triton X-100 and blocked with BSA. The sections or cells were first incubated with primary antibodies recognized as α-smooth muscle actin (α-SMA), and then with the secondary antibody (Beyotime Institute of Biotechnology). Nuclei were stained with DAPI solution (Roche Diagnostics) in the dark and α-SMA was stained in green. Images were taken under an Olympus microscope (magnification, x200; Tokyo, Japan).

### Western blot analysis

Proteins in renal tissue and NRK-52E cells were extracted using RIPA buffer (Beyotime, Shanghai, China). A BCA protein detection Kit (Beyotime, Shanghai, China) was used to measure the protein concentrations in accordance with the manufacturer’s protocol. Proteins were separated by SDS-PAGE. The proteins in the gels were transferred onto polyvinylidene difluoride (PVDF) membranes followed by blockade with 5% skimmed milk. The bolts were then washed and incubated at 4°C overnight with the following primary antibodies: XOD (55,156-1-AP, Proteintech, Wuhan, China), OAT1 (26,574-1-AP, Proteintech, Wuhan, China), OAT3 (ab83789, Cambridge, UK), ABCG2 (27,286-1-AP, Proteintech, Wuhan, China), URAT1 (14,937-1-AP, Proteintech, Wuhan, China), GLUT9 (26,486-1-AP, Proteintech, Wuhan, China), Bcl-2 (26,593-1-AP, Proteintech, Wuhan, China), Bax (50,599-2-Ig, Proteintech, Wuhan, China), cleaved caspase-3 (19,677-1-AP, Proteintech, Wuhan, China), cleaved caspase-9 (Ab2324, Cambridge, UK), fibronectin (15,613-1-AP, Proteintech, Wuhan, China), collagenⅠ(14,695-1-AP, Proteintech, Wuhan, China) and α-SMA (14,395-1-AP, Proteintech, Wuhan, China). Next, the membranes were incubated with secondary antibody coupled with horseradish peroxidase (HRP) anti-rabbit (SA00001-7 L, Proteintech, Wuhan, China) or anti-mouse (SA00001-1, Proteintech, Wuhan, China) at room temperature for 2 h. GAPDH antibody (HRP-60004, Proteintech, Wuhan, China) was used as an internal control. The immunoreactive protein bands were visualized using a SuperSignal West Pico chemiluminescence ECL kit (Pierce). Protein bands were analyzed using ImageJ software (National Institutes of Health).

### Statistical analysis

All data were expressed as mean ± standard deviation. The statistics were analyzed and graphed using GraphPad Prism software (Version 7.0, USA). Student’s t-test was used to perform group comparisons. Statistical differences among multiple comparisons were evaluated by one-way analysis of variance with a post hoc Tukey’s test. P-values of <0.05 were taken as statistically significant.

## Results

### WFA attenuates kidney damage and improves renal functions in PO-induced hyperuricemia mice

**Figure 1.**
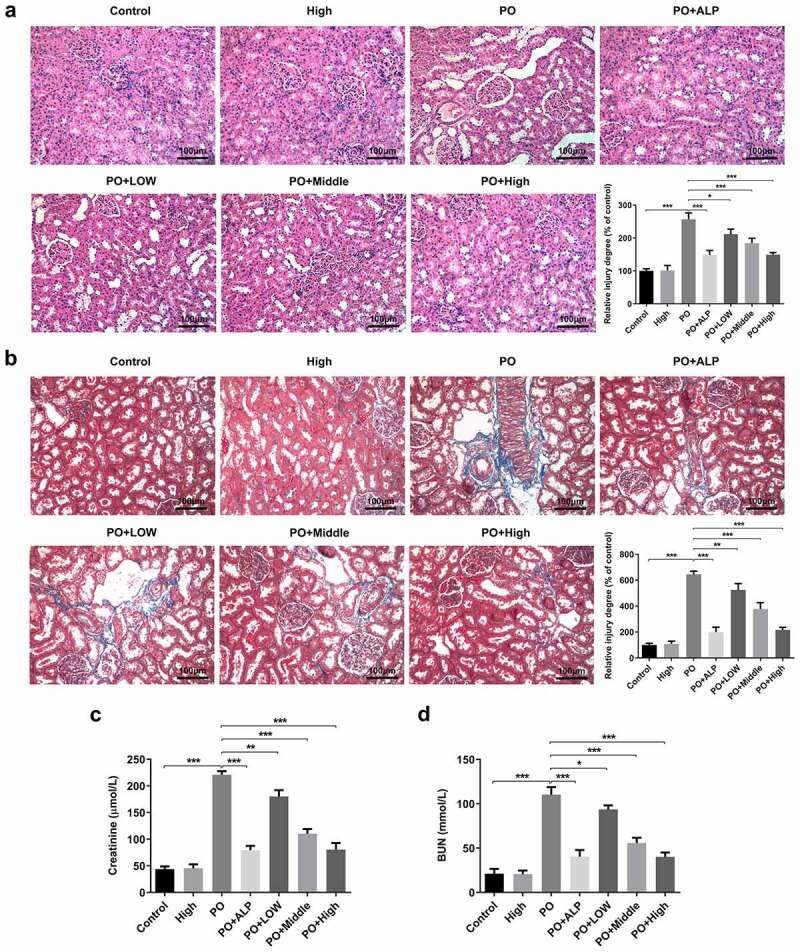
WFA prevented kidney damage and kidney function in PO-induced hyperuricemic mice. (a) Representative H&E images and quantification showing the inhibitory effect of WFA on pathological changes. (b) Representative Masson trichrome staining images and quantification showing the impact of WFA on pathological changes. The levels of (c) creatinine, and (d) BUN were examined by means of kits. N = 10 in each group. Data were expressed as mean ± standard deviation. *P < 0.05, **P < 0.01 and ***P < 0.001. WFA, Withaferin A; PO, potassium oxonate; ALP, Allopurinol; H&E, Hematoxylin & Eosin; BUN, blood urea nitrogen

PO was used to establish the hyperuricemic mouse model as described previously [16]. After treatment with LOW (3 mg/kg), Middle (5 mg/kg) and High (10 mg/kg) doses of WFA or ALP (65 mg/kg), we evaluated the protective effect of WFA on kidney damage in PO-induced hyperuricemic mice. Results from H&E staining and Masson trichrome staining displayed in Figure 1(a,b) showed hypertrophic glomerulus, tubular dilation and increased amount of collagen content in hyperuricemic mice by contrast with the control group, indicating severe glomerular and tubular injury. Of note, WFA treatment dose-dependently improved glomerular and tubular structures. Subsequently, the levels of serum creatinine and BUN were examined to determine whether WFA improves kidney function in PO-induced hyperuricemic mice. As exhibited in Figure 1(c,d), serum creatinine and BUN in PO-treated mice were markedly elevated compared with those in the control group. The group with the highest dose of WFA also exhibited the most elevated level of serum creatinine and BUN. Concurrently, WFA showed no significant effect in normal control mice. These results suggest that WFA does protect against kidney damage and improve renal functions in PO-induced hyperuricemic mice.

### WFA decreases renal fibrosis in PO-induced hyperuricemic mice

**Figure 2.**
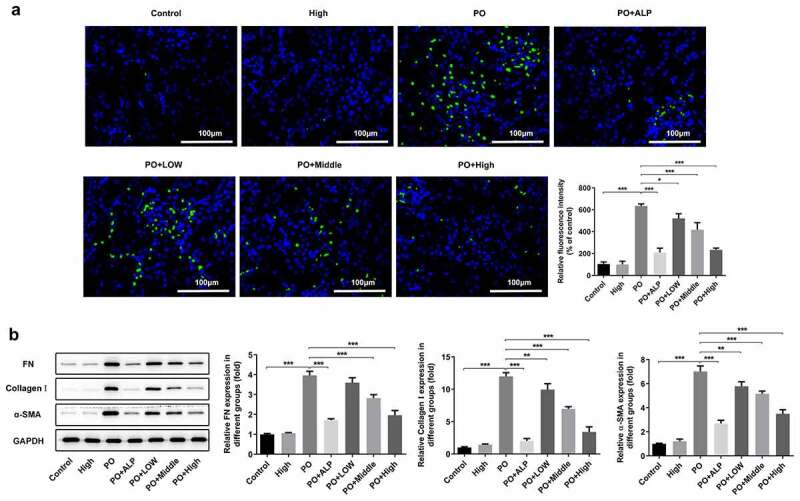
WFA inhibited the fibrosis of kidney tissues in PO mouse model. (a) The expression of α-SMA was determined using immunofluorescence assay. (b) The expression of FN, collagen1 and a-SMA was determined using western blotting. N = 10 in each group. Data were expressed as mean ± standard deviation. *P < 0.05, **P < 0.01 and ***P < 0.001. WFA, Withaferin A; PO, potassium oxonate; α-SMA, α-smooth muscle actin; FN, fibronectin

To investigate the effects of WFA on renal fibrosis in PO-induced hyperuricemic mice, western blot analysis was performed to determine the expression of fibrosis-related proteins in renal tissues. As exhibited in Figure 2(a), the expression of α-SMA detected by immunofluorescence assay was markedly enhanced in the PO group relative to the control group. Notably reduced α-SMA expression was observed after WFA administration, with WFA 10 mg/kg group presenting the most significant inhibitory effect. Consistently, results from western blot analysis demonstrated that WFA dose-dependently controlled the upregulated expression of FN, collagenⅠand α-SMA in the kidney tissues of PO-induced hyperuricemic mice (Figure 2(b)). These findings collectively imply that WFA decreases renal fibrosis in PO-induced hyperuricemic mice.

### WFA inhibits apoptosis in PO-induced hyperuricemic mice

**Figure 3.**
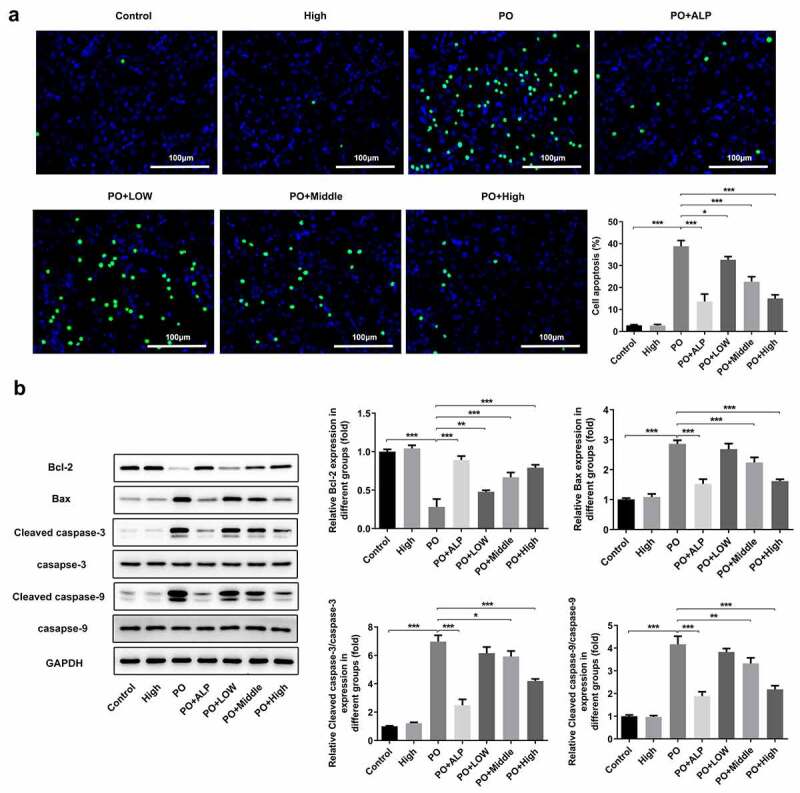
WFA suppressed cell apoptosis of kidney tissues in PO mouse model. (a) Cell apoptosis was examined using TUNEL staining. (b) The levels of apoptosis-related proteins including Bcl-2, Bax, cleaved caspase-3 and cleaved caspase-9 was tested by western blotting. N = 10 in each group. Data were expressed as mean ± standard deviation. *P < 0.05, **P < 0.01 and ***P < 0.001. WFA, Withaferin A; PO, potassium oxonate; ALP, Allopurinol; TUNEL, Terminal-deoxynucleoitidyl Transferase Mediated Nick End Labeling

Furthermore, the effect of WFA on cell apoptosis in PO-induced hyperuricemic mice was evaluated using TUNEL staining. Figure 3(a) showed that the apoptosis level was remarkably elevated in PO-treated group in comparison to that in the control group, which was, however, dose-dependently reversed by WFA administration. It can be observed in Figure 3(b) that the level of anti-apoptosis protein Bcl-2 was dramatically downregulated whereas the expression of pro-apoptosis proteins Bax, cleaved caspase-3 and cleaved caspase-9 was conspicuously upregulated in the kidney tissues of mice in PO group. As expected, these expression trends were all notably turned to the opposite by WFA treatment, suggesting that WFA reduces cell apoptosis in PO-induced hyperuricemic mice.

### WFA reverses the changes in the expression of transportation proteins in PO-induced hyperuricemic mice

**Figure 4.**
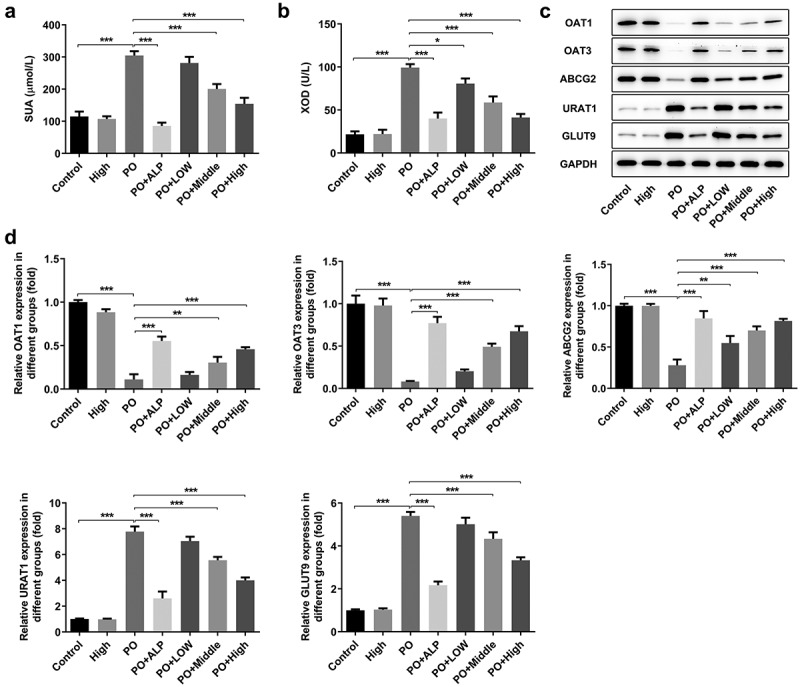
WFA reversed the changed expression of transportation protein in kidney tissues of PO mouse model. The content of (a) UA and the activity of (b) XOD were measured using kits. (c and d) Western blot analysis was employed to evaluate the level of transportation protein. N = 10 in each group. Data were expressed as mean ± standard deviation. *P < 0.05, **P < 0.01 and ***P < 0.001. WFA, Withaferin A; PO, potassium oxonate; ALP, Allopurinol; UA, uric acid; XOD, xanthine oxidase; OAT, organic anion transporter; ABCG2, ATP-binding cassette subfamily G member 2; URAT1, urate reabsorptive transporters urate transporter 1; GLUT9, and glucose transporter 9

It has been corroborated that serum UA plays a vital role in PO-induced hyperuricemia. Therefore, we explored whether WFA affected UA level in our hyperuricemic mouse model. As shown in Figure 4(a), serum UA level was markedly increased in PO-treated mice compared with mice in the control group. Noteworthily, low dose of WFA showed no obvious inhibitory or stimulatory effect on serum UA level. However, a higher dose of 7 to 10 mg/kg of WFA notably reduced the level of UA in contrast with the PO group. XOD is known to be implicated in increased UA level in PO-induced hyperuricemic mice. Therefore, we conducted experiments to investigate whether WFA regulates the activity of XOD in serum of mice with hyperuricemia. Results shown in Figure 4(b) revealed significantly increased XOD activity in the model group relative to the control group, which was restrained by WFA in a dose-dependent way. Subsequently, the levels of URAT1 and GLUT9 which are responsible for renal reabsorption and that of ABCG2, OAT1, and OAT3 which can facilitate renal secretion were examined using western blotting. As what is observable in Figure 4(c,d), the expression of OAT1, OAT3, and ABCG2 was dramatically downregulated in PO-induced hyperuricemic mice. By contrast, the levels of URAT1 and GLUT9 were markedly elevated in the model group compared with the control group. Of note, these changes were significantly inhibited after WFA treatment. It can be concluded by the above observations that WFA reverses the changed expression of renal transporters in PO-induced hyperuricemic mice.

### WFA alleviates UA-induced fibrosis in NRK-52E cells

**Figure 5.**
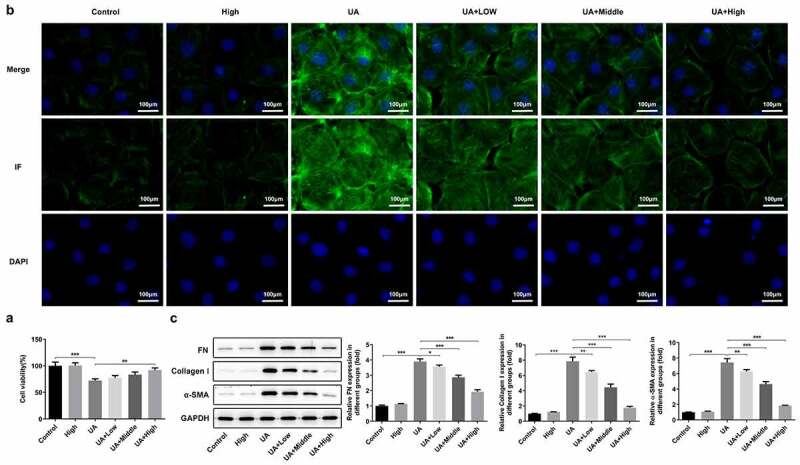
WFA prevented UA-induced fibrosis in NRK-52E cells. (a) Cell viability was assessed with a CCK-8 assay. (b) The level of α-SMA was determined using immunofluorescence assay. (c) Western blotting was employed to assess the expression of FN, collagen1 and a-SMA. Data were presented as the mean ± standard deviation of three independent experiments. **P < 0.01 and ***P < 0.001. WFA, Withaferin A; UA, uric acid; α-SMA, α-smooth muscle actin; FN, fibronectin

Subsequently, whether WFA prevents UA-induced expression of fibrosis-related genes in NRK-52E cells was later explored as well in this study. It was found that UA stimulation remarkably inhibited viability of NRK-52E cells as comparison to the control (Figure 5 (a)). WFA intervention after UA induction significantly enhanced cell viability in a dose-dependent manner, while treatment of WFA alone in the absence of UA induction did not affect the viability of NRK-52E cells. Moreover, notably increased α-SMA expression was noticed in the UA induction group, which was remarkably inhibited by WFA treatment (Figure 5(b)). Similarly, UA significantly upregulated the expression of FN and collagenⅠin NRK-52E cells. Notably, WFA attenuated the impact of UA on the levels of the above-mentioned fibrosis-associated proteins in NRK-52E cells (Figure 5(c)). Taken together, the above findings prove that WFA inhibits UA-induced fibrosis in renal tubular cells.

### WFA blocks UA-induced apoptosis in NRK-52E cells

**Figure 6.**
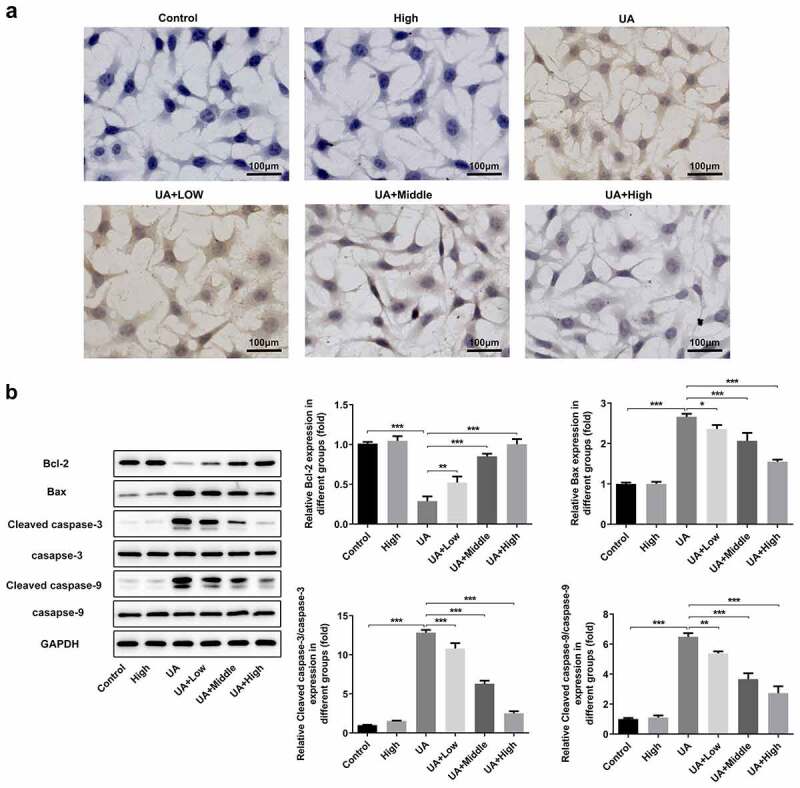
WFA blocks UA-induced apoptosis in NRK-52E cells. (a) Apoptosis of NRK-52E cells induced by UA was evaluated using TUNEL staining. (b) The levels of apoptosis-associated proteins was tested with western blot analysis. Data were presented as the mean ± standard deviation of three independent experiments. *P < 0.05, **P < 0.01 and ***P < 0.001. Withaferin A; UA, uric acid; TUNEL, Terminal-deoxynucleoitidyl Transferase Mediated Nick End Labeling.**Highlights.**

Finally, we researched whether WFA could attenuate UA-induced cell apoptosis in NRK-52E cells. Figure 6(a) showed the result from TUNEL staining assay, demonstrating UA exposure noticeably induced cell apoptosis in NRK-52E cells. In consistent with the results from TUNEL staining, western blot assay showed that UA markedly upregulated the expression of pro-apoptosis genes including Bax, cleaved caspase-3 and cleaved caspase-9, accompanied by decreased level of anti-apoptosis Bcl-2 (Figure 6(b)). In a word, these results suggest that WFA effectively prevents UA-induced cell apoptosis and regulates the expression of apoptosis-related genes in NRK-52E cells.

1. WFA effectively attenuates the PO-induced kidney histological damage.2. WFA alleviates PO-induced renal tissues fibrosis and apoptosis.3. WFA inhibits the expression of transportation proteins in PO-induced renal tissues.4. WFA improves UA-induced fibrosis and apoptosis in NRK-52E cells.

## Discussion

WFA is a steroidal lactone derived from Withania somnifera, a kind of plant commonly used in traditional ayurvedic medicine, and it has been reported to ameliorate unilateral ureteral obstruction-induced renal injury in mice [17]. Existing study has shown that WFA reverses liver fibrosis induced by bile duct ligation via inhibiting extracellular matrix deposition [18]. *In vivo* and *in vitro* experiments have also proven that WFA can improve pulmonary fibrosis through regulation of the reciprocity among fibrotic, matricellular proteins and cytokines [19]. Additionally, emerging evidence suggests that WFA has a neuroprotective effect on traumatic brain injury most likely through modulating the activation of microglia cells and arresting the apoptosis of vascular endothelial cells [20]. The recent study has suggested that WFA protects against unilateral ureteral obstruction-induced renal injury via inhibiting endoplasmic reticulum stress-associated apoptosis, inflammation, and fibrosis [21]. In the present study, we explore the possible protective effect of WFA on renal injury induced by hyperuricemia *in vivo* and *in vitro*. Results demonstrated that WFA alleviated renal damage and improved kidney function in mice with hyperuricemia. Furthermore, WFA significantly inhibited renal fibrosis and apoptosis. Further mechanistic study showed that WFA significantly inhibited the increased XOD in the kidney of PO-induced hyperuricemic mice. Moreover, WFA significantly reversed the decreased levels of transporters responsible for secretion including OAT1, OAT3, ABCG2, and the increased expression of transporters including URAT1 and GLUT9 in PO-induced hyperuricemic mice. Finally, our study at the cellular level exhibited that WFA significantly inhibited UA-induced cell apoptosis and fibrosis-related protein expression including that of fibronectin, collagen, and α-SMA.

PO is most frequently used to set up animal models of hyperuricemia via suppressing uricase that converts UA to allantoin [22]. In this study, we successfully established PO-induced hyperuricemic mouse model, and evaluated the protective effect of WFA. Our results have shown that WFA effectively protected against kidney damage and improved kidney function in PO-induced hyperuricemic mice. Besides, the increased expression and the activity of XOD are also responsible for hyperuricemia in PO-induced hyperuricemic mice [16]. For example, it has been shown that ChondroT improves kidney function by downregulation of XOD in PO-induced hyperuricemic mice [16]. Another study demonstrated that curcumin attenuates hyperuricemia and kidney inflammation via inhibition of XOD activity in PO-induced hyperuricemic mice [23]. In consistent with these studies, the present study corroborated that WFA is effective in preventing a rise in the level of XOD in the kidney of PO-induced hyperuricemic mice.

Hyperuricemia occurs when the balance between production and complicated course of UA handing in renal tubules and intestines is disrupted [24]. It has been demonstrated that almost all urate transport occurs in the proximal tubule, where there is only a minimal amount of distal tubule transport, as evidenced by some other studies [25,26]. It is known that kidney is the main organ for excretion of approximately two-thirds of the total UA [27]. The process of UA reabsorption is completed through URAT1 and GLUT9 after glomerular filtration, after which UA will be secreted into intestinal tract or other tissues through ABCG2, OAT1, and OAT3 [28]. We observed elevated expression of URAT1 and GLUT9, which are two proteins responsible for renal reabsorption, and notably decreased expression of ABCG2, OAT1, and OAT3, which are three proteins responsible for renal secretion. This finding is consistent with that of a previous study where the expression of UA transporter was found to be responsible for hyperuricemia [6]. Of note, the expression trends of these UA transporter genes were remarkably inhibited by WFA, which might explain how WFA exerts its protective effect.

It has been shown that UA could induce the apoptosis of renal tubular cells, thereby leading to renal fibrosis and kidney damage [29,30]. In compliance with these studies, this study showed that UA markedly increased the levels of α-SMA, fibronectin and collagen, and that it dramatically elevated cell apoptosis in NRK-52E cells. Notably, all above-mentioned changes induced by UA were notably abrogated by WFA. These findings suggest that WFA may exert its protective effect by directly acting on renal tubular cells.

### Conclusion

Taken together, the current study demonstrates that WFA reduces UA and thereby improves kidney function via regulation of XOD and transporter-related genes in renal tubular cells. Our research findings may provide a theoretical basis for the application of WFA to the prevention and treatment of kidney damage in hyperuricemia. Whether there is a protective effect of WFA on inflammatory response in hyperuricemia-induced kidney injury will be investigated in the following experiments, which is a limitation of the present study.

## Data Availability

All data generated or analyzed during this study are included in this published article.
